# Identification and functional characterization of D-fructose receptor in an egg parasitoid, *Trichogramma chilonis*

**DOI:** 10.1371/journal.pone.0217493

**Published:** 2019-06-19

**Authors:** Jianbai Liu, Han Wu, Jiequn Yi, Dingxin Jiang, Guren Zhang

**Affiliations:** 1 State Key Laboratory for Biocontrol, Sun Yat-Sen University, Guangzhou, China; 2 Guangdong Engineering Research Center for Pesticide and Fertilizer, Guangdong Bioengineering Institute (Guangzhou Sugarcane Industry Research Institute), Guangzhou, China; 3 Key Laboratory of Natural Pesticide and Chemical Biology, Ministry of Education, Laboratory of Insect Toxicology, South China Agricultural University, Guangzhou, People’s Republic of China; Institut Sophia Agrobiotech, FRANCE

## Abstract

In insects, the gustatory system has a critical function not only in selecting food and feeding behaviours but also in growth and metabolism. Gustatory receptors play an irreplaceable role in insect gustatory signalling. *Trichogramma chilonis* is an effective biocontrol agent against agricultural insect pests. However, the molecular mechanism of gustation in *T*. *chilonis* remains elusive. In this study, we found that *T*. *chilonis* adults had a preference for D-fructose and that D-fructose contributed to prolong longevity and improve fecundity. Then, We also isolated the full-length cDNA encoding candidate gustatory receptor (TchiGR43a) based on the transcriptome data of *T*. *chilonis*, and observed that the candidate gustatory receptor gene was expressed from the larval to adult stages. The expression levels of *TchiGR43a* were similar between female and male. A *Xenopus* oocyte expression system and two-electrode voltage-clamp recording further verified the function analysis of TchiGR43a. Electrophysiological results showed that TchiGR43a was exclusively tuned to D-fructose. By the studies of behaviour, molecular biology and electrophysiology in *T*. *chilonis*, our results lay a basic fundation of further study on the molecular mechanisms of gustatory reception and provide theoretical basis for the nutritional requirement of *T*. *chilonis* in biocontrol.

## Introduction

Animals evolved a gustatory system that possesses the ability to detect and distinguish different taste stimuli in their living environments. The taste sensory system, which identifies and evaluates potential foods by discriminating between nutrients that benefit feeding behaviour, growth, and metabolism and harmful or even toxic compounds that are adverse to survival, is essential for most animals, ranging from flies to humans [1, 2].

In insects, the taste sensory system has a significant effect on feeding, courtship, mating and ovipositing [3]. Taste stimuli from the environment are recognized and assessed by multiple sets of gustatory receptors (GRs) and gustatory receptor neurons (GRNs) housed in sensilla scattered on different tissues, including the labial palps, labellum, antennae, tarsi, legs, wings and pharyngeal sense organs [4–7]. Most sensilla house four gustatory neurons and one mechanosensory neuron. Of the gustatory neurons, one is the “sugar” neuron sensitive to sugars such as sucrose, glucose, fructose and other sugars; one is the “salt” neuron sensitive to salts; one is the “bitter” neuron sensitive to aversive compounds such as quinine, chloroquine, caffeine and strychnine; and one is the “water” neuron sensitive to pure water [8–11]. Previous study shows that insect gustatory receptors have a special seven-transmembrane domain with an extracellular C-terminus and an intracellular N-terminus, which possess a reverse topology that is different from the typical G-protein coupled receptors (GPCRs) [12, 13]. Gustatory receptors are diverse and complex, which differ from olfactory receptors (ORs), with heterodimeric receptors (OR and ORCO) that can work; thus, an understanding of the mechanism of gustatory sensory systems is necessary for identifiying the function of the gustatory receptors.

Previous studies of genomes and transcriptome analyses show that different GR genes exist in insects, including *Drosophila melanogaster* (60), *Manduca sexta* (12), *Apis mellifera* (12), *Linepithema humile* (96), *Bombyx mori* (69) and *Anopheles gambiae* (76) [5, 14–18]. To date, most research related to gustatory receptors has been conducted primarily on the model organism *D*. *melanogaster*. The function of these GRs in *D*. *melanogaster* is in sensing sweet or bitter chemical compounds. Some gustatory receptors in *D*. *melanogaster* are required to sense trehalose, others are responsible for sensing fructose, sucrose, glucose, and maltose [8, 10, 19–24], while there are also a number of receptors have the function to detect aversive compounds, such as caffeine, umbelliferone, L-canavanine and strychnine [25–33]. Moreover, the function of gustatory receptors has also been studied in a few other insects. In *B*. *mori*, some taste receptors have the function to sense fructose and inositol [3, 13, 24, 34]. Additionally, in *A*. *mellifera*, several gustatory receptors show sensitivity to sucrose and fructose [35–37]. However, studies of molecular mechanisms of the gustatory system in Hymenoptera insect species, particularly parasitoid wasps, remain scarce.

Fructose is one of the most common sugars in floral nectars and honeydew in nature. At the same time, floral nectars and honeydew also contain trace amounts of other sugars, such as mannose (monosaccharide), maltose (disaccharide) and melezitose (oligosaccharide). Fructose can stimulate parasitoids to eat [38], and affects their lifespan and fecundity [39–42], at the same time, the nutritional status of parasitoids greatly influences their behaviors [43]. Both practically and theoretically, Sugar intake is very important for insect survival in the wild, and can extend their life span and increase their fertility [44, 45].

The egg parasitoid *Trichogramma chilonis* (Ishii) (Hymenoptera: Trichogrammatidae), is one of the most successful biological control agents of agricultural and forest insect pests. In China, *T*. *chilonis* is often utilized to control Lepidoptera pests [46–48]. To date, research on *T*. *chilonis* has primarily focused on mass rearing and improving the parasitism rate [49–51], while there are few studies on biochemistry and molecular biology, since the size of *T*. *chilonis* is too small (0.2~0.4 mm) to many experimental operations. Gustation of *T*. *chilonis* is extremely vital in foraging, mating, ovipositing and other physiological behaviours. The characterization of gustatory receptors in *T*. *chilonis* may increase the understanding of the molecular mechanisms of feeding behaviour and host seeking and suggest novel strategies for application in biological control.

In a previous transcriptome study of *T*. *chilonis*, we found a potential gustatory receptor gene, but its function and potential ligands are still unknown, as are their effects on life-history traits [52]. In this study, we first conducted tests to clarify the behavioural and physiological effects of D-fructose on *T*. *chilonis* adults. Then, we cloned the gustatory receptor candidate gene, *TchiGR43a*, from the transcriptome of *T*. *chilonis* and confirmed the expression patterns of the candidate gene in different life stages and different genders of *T*. *chilonis* by qRT-PCR. Last, we identified the function and ligands of TchiGR43a by using a *Xenopus laevis* oocyte expression system. This study will provide a solid foundation for further research on gustatory reception of *T*. *chilonis* and a theoretical basis for the application of this egg parasitoid in biological control programs.

## Materials and methods

### Insects

Adult *T*. *chilonis* and the host *Corcyra cephalonica* (Stainton) eggs were originally obtained from the Plant Protection Research Institute, Guangdong Academy of Agricultural Sciences, People’s Republic of China. All *C*. *cephalonica* eggs were sterilized by ultraviolet radiation. The parasitized *C*. *cephalonica* eggs were reared at 25±1°C with 75±5% relative humidity and 14 (h) L: 10 (h) D photoperiod. After ten generations, the *T*. *chilonis* that emerged from the *C*. *cephalonica* eggs were used in the experiments.

### Chemicals

D-glucose, myo-inositol, D-lactose, D-trehalose, sucrose, D-fructose, D-maltose, D-galactose, L-sorbose, D-mannose, and D-arabinose were obtained from Sigma Chemical Company (St. Louis, MO, USA). All chemicals were analytical grade (>99.5%).

### Behavioural, longevity and fecundity experiments

**Fig 1:**
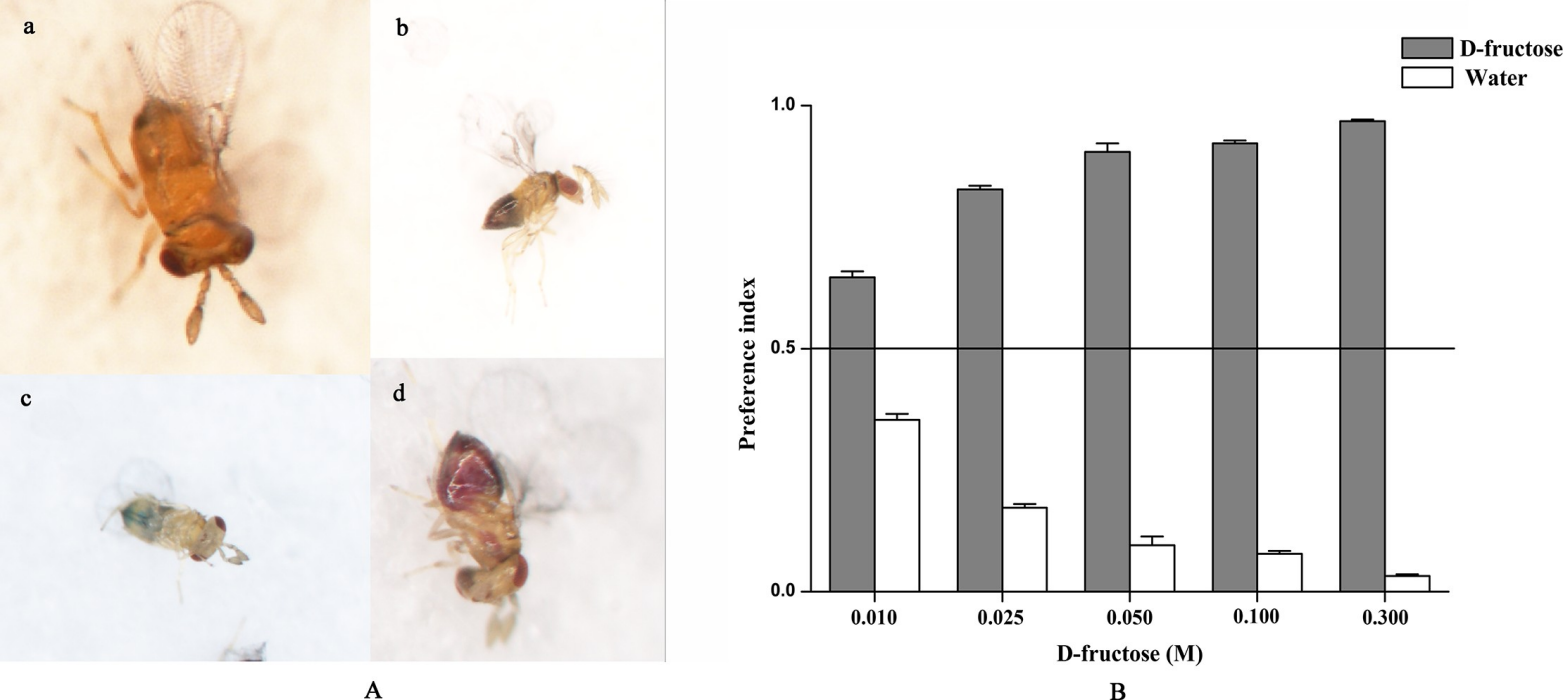
Behavioral preference to D-fructose of *T*. *chilonis*. A: a, *T*. *chilonis* fed nothing; b, *T*. *chilonis* fed both fructose and water; c, *T*. *chilonis* fed water; d, *T*. *chilonis* fed sugar. B: Relative sensitivity of D-fructose was determined by two-choice preference tests. PI values for D-fructose are shown at the following concentrations: 0.010, 0.025, 0.050, 0.100 and 0.300 M every concentration was tested with 50–60 adults. Error bars indicate SEMs from the analysis of three replications (P < 0.05).

To understand the ability to detect fructose, adults of *T*. *chilonis* were deprived of food (only supplied with water) for 24 hours. Then, 50~60 parasitoids were randomly selected into a glass tube that contained fructose solution colored with sulforhodamine B (0.2mg/mL) (red dye) and distilled water coloured with brilliant blue FCF (0.125mg/mL) (blue dye), kept at 25±1°C with 75±5% relative humidity for 2 hours. The results were verified by using a stereomicroscope, parasitoids that fed on fructose had their bellies dyed red, parasitoids that fed on distilled water had their bellies dyed blue and parasitoids that fed on both solutions had their bellies dyed purple. The preference index (PI) for D-fructose was calculated using the following formula: PI = (N ^Red^ + 0.5 N ^Mix^) / (N ^Red^ + N ^Blue^ + N ^Mix^) [53], where N ^Red^, N ^Blue^ and N ^Mix^ represent the number of *T*. *chilonis* coloured red, blue and purple (Fig 1A), respectively. PI ≤ 0.5 indicates no preference and PI of 0.5–1.0 indicates a preference. The attractiveness was induced by D-fructose with a set of concentrations (0.010 M, 0.025 M, 0.050 M, 0.100 M and 0.300 M). This experiment was tested in triplicate.

For adult *Trichogramma* species, longevity and fecundity play crucial roles that affect oviposition and even the mass culture. To further evaluate the effects of D-fructose on *T*. *chilonis*, we investigated the longevity and fecundity of this parasitoid in conditions with D-fructose and without D-fructose. For the longevity experiments, 90 females were divided evenly into three groups, and these groups were fed a 0.050 M D-fructose solution, distilled water and nothing, respectively, D-fructose solution and distilled water were refreshed every 8 hours. The treatments feeding with distilled water and nothing were set as controls. In every group, *T*. *chilonis* female adults were individually introduced into a glass vial (diameter 2.2 cm, height 10 cm) that contained a piece of cardboard carrying approximately 1000 UV-sterilized *C*. *cephalonica* eggs and a droplet (10 μL) of D-fructose solution. Vials were closed with cotton gauze and kept in an environmental chamber (25±1°C, 75±5% relative humidity, 14 (h) L:10 (h) D photoperiod). In every group, each female is an experiment replication. Dead parasitoids were checked daily using a stereomicroscope.

For the fecundity experiments, 180 females were divided evenly into three groups, and these groups were fed a 0.050 M D-fructose solution, distilled water and nothing, respectively. Every group has 60 females, each female *T*. *chilonis* adult was individually introduced into a glass vial (each female was in a separate glass vial). In every group, each female is an experiment replication. Feeding with distilled water and nothing were set as controls. The glass vial (diameter 2.2 cm, height 10 cm) contained a piece of cardboard carrying approximately 1000 UV-sterilized *C*. *cephalonica* eggs and a droplet (10 μL) of a D-fructose solution or distilled water. Vials were closed with cotton gauze and kept in an environmental chamber (25±1°C, 75±5% relative humidity, 14 (h) L:10 (h) D photoperiod). *C*. *cephalonica* eggs were refreshed every day, and the foods (D-fructose solution, distilled water and nothing) were refreshed every 4 hours. The parasitized *C*. *cephalonica* eggs were cultured in the same environment, and the number of parasitized eggs was recorded when the host eggs turned dark.

### Cloning of the candidate gustatory receptor of *T*. *chilonis*

To understand the molecular mechanism of the candidate gustatory receptor gene in *T*. *chilonis*, we first used the transcriptome data of *T*. *chilonis* from our previous work (SRA accession number: SRP137064) [52].

Total RNA was extracted from adult *T*. *chilonis* using TRIzol reagent (Invitrogen, USA) according to the manufacturer's instructions. The first-strand complementary DNA (cDNA) was synthesized using a PrimeScript RT reagent Kit with gDNA Eraser (Takara, Kyoto, Japan). Templates for 5’ and 3’ RACE were prepared using a SMART RACE cDNA Amplification Kit (Clontech, Mountain View, CA, USA) according to the manufacturer's instructions. Primers (S1 Table) were designed based on the nucleotide sequences. Nested polymerase chain reaction (PCR) was performed to obtain the 5’-end/3’-end sequence with primer pairs. PCR products were cloned and then sequenced by Invitrogen (Shanghai).

### Phylogenetic analysis

The full-length protein sequence of the putative gustatory receptor gene in our study was phylogenetically analysed with the homologues from Diptera (*D*. *melanogaster*), Lepidoptera (*B*. *mori*) and Hymenoptera (*T*. *pretiosum*, *N*. *vitripennis*, *Apis mellifera*, *C*. *floridanum*, *Cephus cinctus*, *Orussus abietinus*, *Pseudomyrmex gracilis* and *Athalia rosae*). The phylogenetic tree was constructed using the neighbour-joining method with 1000 bootstrap replications in MEGA 6.06.

### qRT-PCR analysis of *TchiGR43a*

Total RNA of *T*. *chilonis* was extracted using the TRIzol method (Taraka, Japan). To obtain the first-strand cDNAs, 1 μg of total RNA was used for reverse transcription in a reaction system with a total volume of 20 μL, according to the manufacturer's instructions (PrimeScript RT Reagent Kit, TaKaRa, Japan). qRT-PCR was performed using LightCycler480 SYBR-Green I Master (Roche Diagnostics, Basel, Switzerland) and run on the LightCycler480 Real-time PCR system (Roche Diagnostics Ltd). Each reaction was conducted in a reaction system with a total volume of 10 μL with 1 μL of cDNA (2 ng/μL), 5 μL of SYBR Green I Master (LightCycler480 SYBR Green I Master, Roche Diagnostics Ltd., Lewes, UK), 0.5 μL/primer, and 3 μL of ddH_2_O. The qRT-PCR was conducted using the following programme: denaturation at 95°C for 5 min, followed by 40 cycles of 5 s at 95°C, 20 s at 60°C, and 20 s at 72°C. *gapdh* was the internal reference gene. Each gene was tested in triplicate, and the experiments were conducted on three biological replicates. The relative expression levels of the genes normalized to the internal control gene, were calculated using the 2^-ΔΔCt^ method [54]. Analysis of relative gene expression data used a real time quantitative PCR and the 2^-ΔΔCt^ method.

### Functional characterization of TchiGR43a

To identify the function of the candidate D-fructose receptor of *T*. *chilonis*, we examined electrophysiological responses of *Xenopus* oocytes expressing *TchiGR43a* to 11 sugars at the concentration of 0.100 M. The full-length open reading frame sequence of TchiGR43a cDNA was amplified by RT-PCR, and first cloned into pMD 19-T vectors (Taraka, Japan). Then, the sequence was subcloned into a pCS2+ vector. The cRNA of TchiGR43a was synthesized from linearized modified pCS2+ vectors with a mMESSAGE mMACHINE SP6 Transcription Kit (Ambion, Austin, TX, USA), according to the manufacturer's instructions. Mature *Xenopus laevis* oocytes were digested and isolated by 2.0 mg/mL of collagenase type IA (Sigma-Aldrich) in a solution (96.0 mM NaCl, 2.0 mM KCl, 1.0 mM MgCl _2_, 5.0 mM HEPES, 2.5 mM Na-pyruvate, pH 7.5) without Ca^2+^ for 15~30 min at room temperature. Then, 50 ng of TchiGR43a cRNA was microinjected into every individual oocyte. The injected oocytes were cultured in culture solution (96.0 mM NaCl, 2.0 mM KCl, 1.0 mM MgCl_2_, 1.8 mM CaCl_2_, 5.0 mM HEPES, 2.5 mM Na-pyruvate, 0.5 mM Theophyline, pH 7.5) at 16°C, and the culture solution was refreshed every day. After 72 hours of culture, a two-electrode voltage clamp with recording solution (96.0 mM NaCl, 2.0 mM KCl, 1.0 mM MgCl_2_, 1.8 mM CaCl_2_, 10.0 mM HEPES, pH 7.5) recorded the injected cells. The two glass electrodes were filled with 3.0 M KCl, and their resistances were kept between 0.2 and 2.0 MΩ. All the signals were collected and amplified by an AxoClamp 900A amplifier (Axon Instruments Inc., Foster City, CA, USA) at a holding potential of -80 mV, low-pass filtered at 50 Hz and digitized at 1 kHz. Data acquisition and analysis were conducted with Digidata 1550A and pCLAMP software (Axon Instruments Inc., Foster City, CA, USA). Dose-response data were analysed with GraphPad Prism 6, and EC_50_ (concentration for 50% of maximal effect) was calculated to show the sensitivity of the receptor to the ligand.

### Data analyses

All results are expressed as the mean±SEM, and the data were analysed using one-way analysis of variance (ANOVA), followed by Duncan’s multiple range test for multiple comparisons. To compare the sensitivity of GR to D-fructose and myo-inosotiol, a t–test was used. Statistical significance was determined at the P<0.05 level. Statistical analyses were performed using the SPSS 19.0 statistical software package.

## Results

### Behaviour al preference of D-fructose

From the tests, *T*. *chilonis* adults showed a significant preference for D-fructose compared with water. To verify the sensitivity of *T*. *chilonis* adults to D-fructose, we calculated the attractive preference index. The PI of D-fructose rised with the increase in concentration of D-fructose (Fig 1B). These data indicated that *T*. *chilonis* has an ability of sensing different concentrations of D-fructose.

### Effect of D-fructose on the longevity and fecundity of *T*. *chilonis* adults

**Fig 2:**
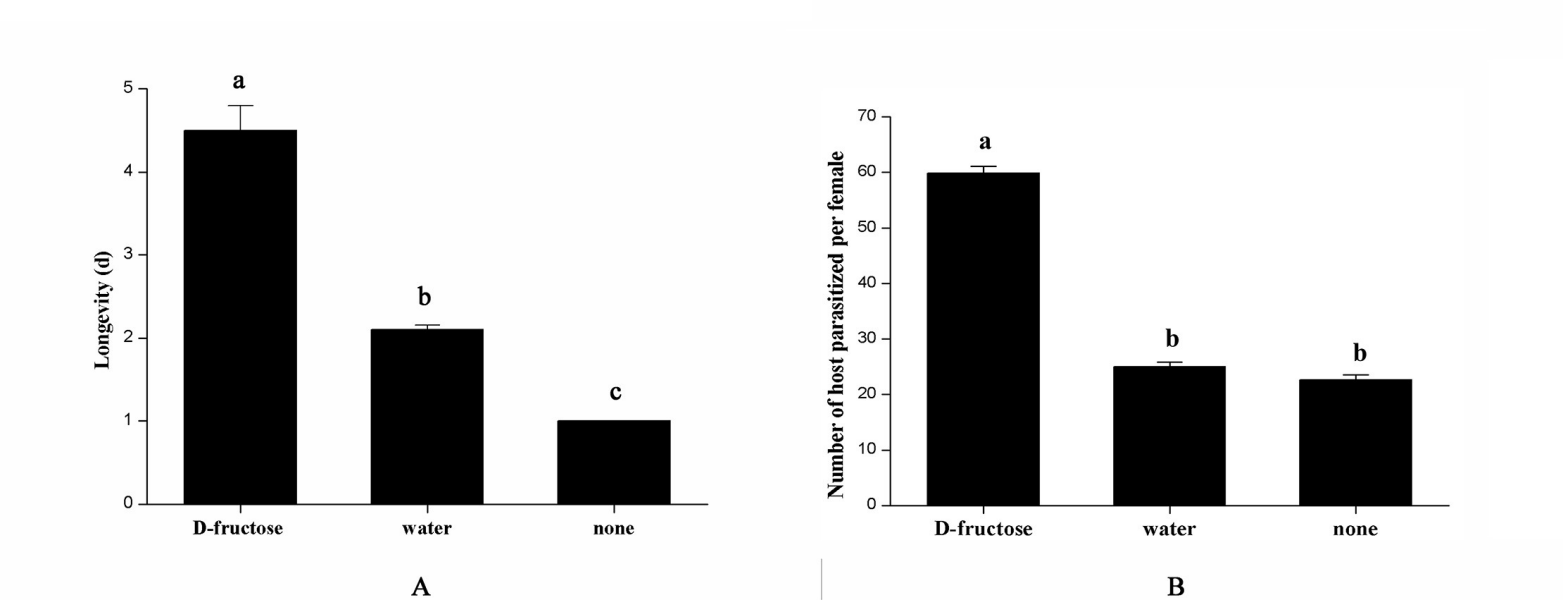
Longevity and fecundity of female *T*. *chilonis* when supplied with D-fructose, water and nothing. (A) Longevity, error bars indicate SEMs from the analysis of 30 replications (P < 0.05).; (B) Fecundity, error bars indicate SEMs from the analysis of 60 replications (P < 0.05).

D-fructose significantly increased the longevity of *T*. *chilonis* adults when compared with the controls (Fig 2A). Between the two controls, adults fed water lived longer than those not fed. The fecundity of *T*. *chilonis* varied significantly between the D-fructose treatment and controls. The 0.050 M D-fructose solutions significantly improved the fecundity (Fig 2B). The fecundity of the two controls was similar. From the experiments, fecundity and longevity of *T*. *chilonis* highly benefited from feeding on D-fructose.

### The candidate gustatory receptors identified from *T*. *chilonis*

To understand the molecular mechanism of taste detection in *T*. *chilonis*, we identified the candidate gustatory receptor gene that might be sensitive to some special tastants based on previous analysis of information from the transcriptome database of *T*. *chilonis* [53]. With RACE PCR, we obtained the full-length cDNA that encoded putative gustatory receptor in *T*. *chilonis*.

**Fig 3:**
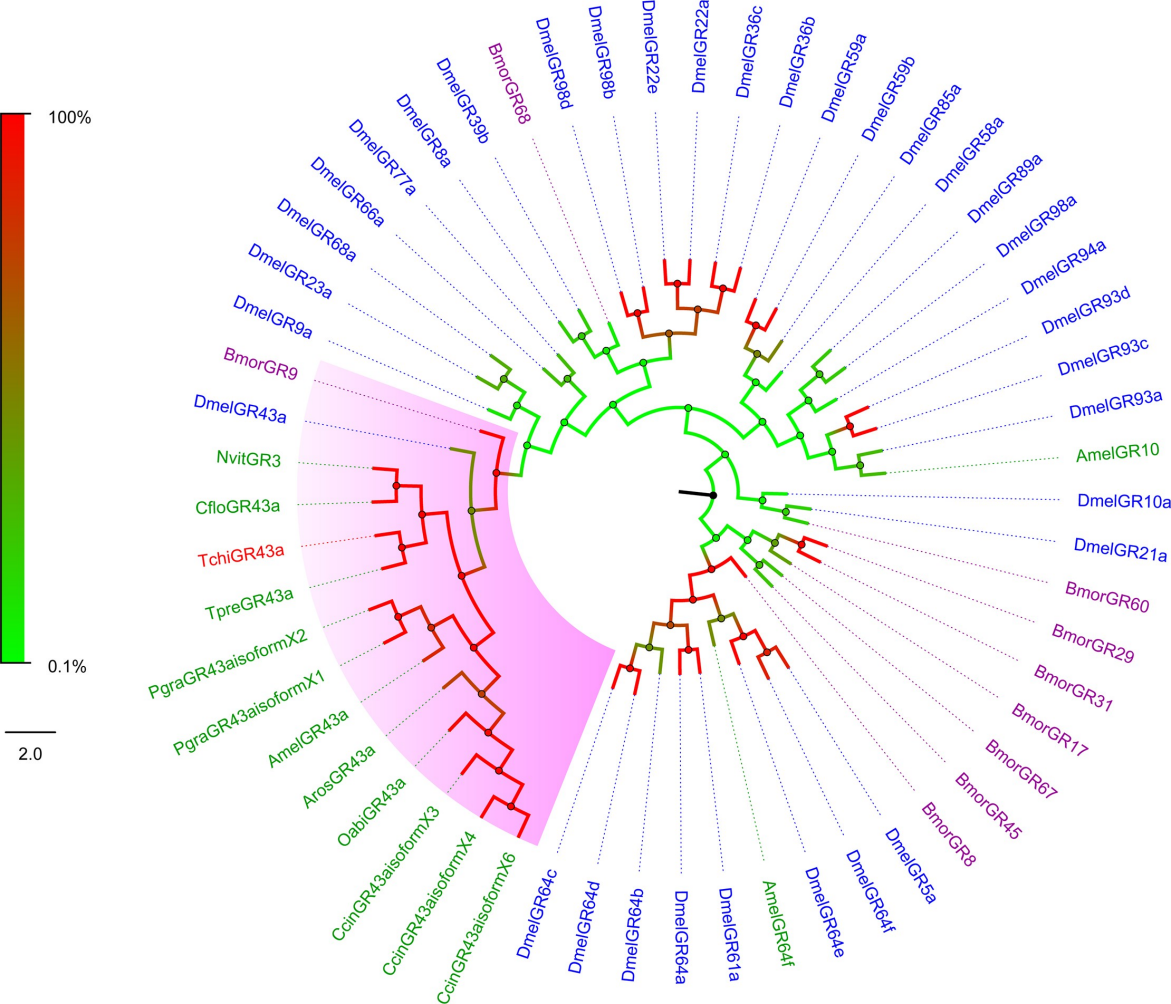
Phylogenetic analysis of putative gustatory receptors of *T*. *chilonis*. The tree was constructed in MEGA6.0 using the neighbor-joining method. TchiGR43a from *T*. *chilonis* are labelled with red, GRs from *D*. *melanogaster* (Diptera) are labelled with blue, GRs from *B*. *mori* (Lepidoptera) are labelled with purple, and GRs from other Hymenoptera insects (*T*. *pretiosum*, *N*. *vitripennis*, *Apis mellifera*, *C*. *floridanum*, *Cephus cinctus*, *Orussus abietinus*, *Pseudomyrmex gracilis* and *Athalia rosae*) are labelled with green.

The full-length cDNA sequence of the candidate *TchiGR* gene was 1930 bp, with the GC content of 54.72% and an open reading frames (ORFs) of 1650 bp that encoded protein sequences of 549 amino acid residues. The candidate TchiGR had an identity higher than 60% with GRs from *Trichogramma pretiosum*, *Nasonia vitripennis* and *Copidosoma floridanum*. To assign putative functions to the candidate *TchiGR* gene, phylogenetic analysis of the TchiGR and GRs from Diptera (*D*. *melanogaster*), Lepidoptera (*B*. *mori*) and Hymenoptera (*T*. *pretiosum*, *N*. *vitripennis*, *Apis mellifera*, *C*. *floridanum*, *Cephus cinctus*, *Orussus abietinus*, *Pseudomyrmex gracilis* and *Athalia rosae*) insects was performed. According to this GR phylogenetic tree, the candidate TchiGR clustered phylogenetically with DmelGR43a, BmorGR9 and the members of a GR43a subclade from Hymenoptera insects (Fig 3). Most of the splits in the phylogenetic tree were strongly supported by high bootstrap values. According to the results of phylogenetic tree analysis and conventions of GR nomenclature, we named the putative gustatory receptor gene *TchiGR43a* (GenBank accession numbers: MH816967).

### Expression patterns of *TchiGRs* of *T*. *chilonis*

**Fig 4:**
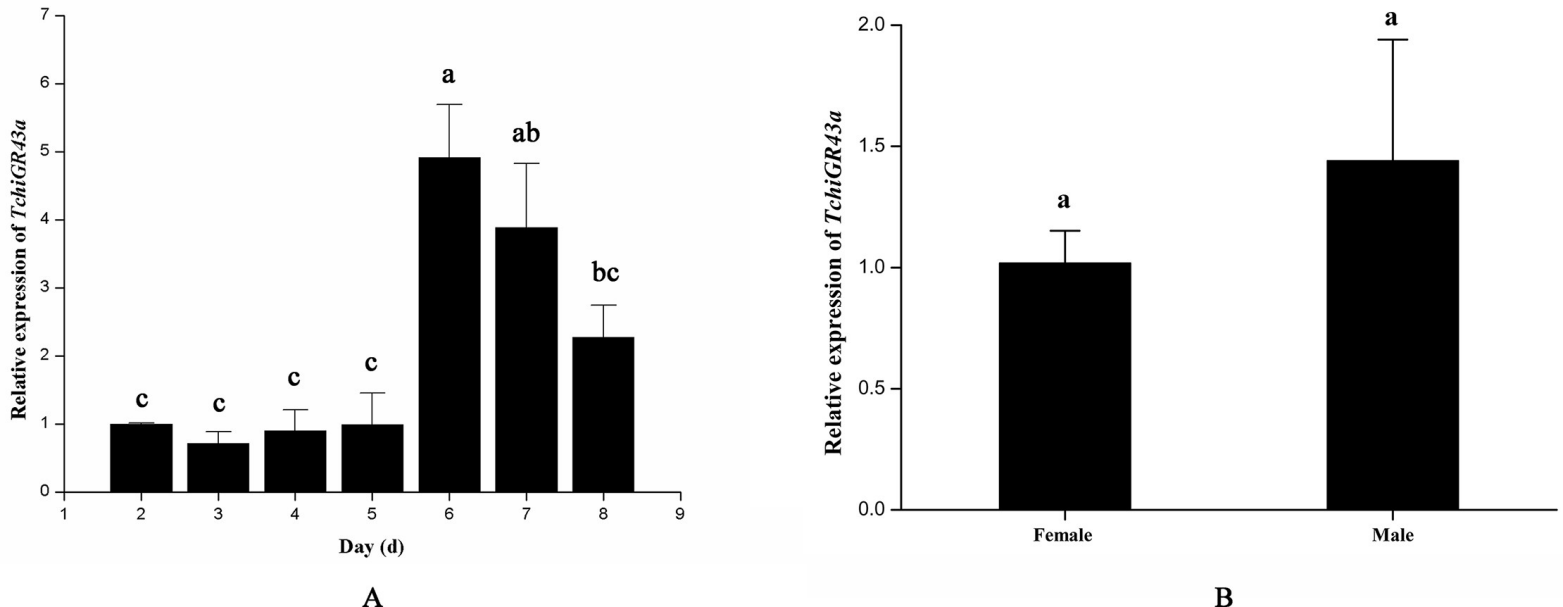
Expression patterns of *TchiGR43a* in *T*. *chilonis*. (A) Relative expression levels of *TchiGR43a* in different developmental stages of *T*. *chilonis* by qRT-PCR analysis. Larval stage: d2, prepupal stage: d3-d4, pupal stage: d5-d8. (B) Relative expression levels of *TchiGR43a* between male and female adult *T*. *chilonis* by qRT-PCR analysis. Error bars indicate SEMs from the analysis of three replications (P < 0.05).

The relative expression levels of the gustatory receptor gene in different developmental stages and in male and female adult *T*. *chilonis* were quantified by qRT-PCR with specific primers (Fig 4). *T*. *chilonis* is a holometabolous insect with several developmental stages, including the 26-hour egg stage, 36-hour larval stage, 48-hour prepupal stage, 84-hour pupal stage and 1-3-day adult stage [55]. The results of qRT-PCR showed that *TchiGR43a* expressed from the larval stage (d2) to prepupal stage (d3-d4) to pupal stage (d5-d8). The expression of the gustatory receptor gene showed a trend in which the expression level of *TchiGR43a* declined from the larval stage to the prepupal stage, then increased from the prepupal stage to the pupal stage and declined again at the late stage of the pupa (Fig 4A). The highest expression level of *TchiGR43a* was detected in the prometaphase of the pupal stage (d6), while the lowest expression level appeared in the prometaphase of the prepupal stage (d3). In adult male and female *T*. *chilonis*, the expression levels of *TchiGR43a* showed a similar pattern (Fig 4B).

### Functional assay of *TchiGR43a* using two-electrode voltage-clamp recording

**Fig 5:**
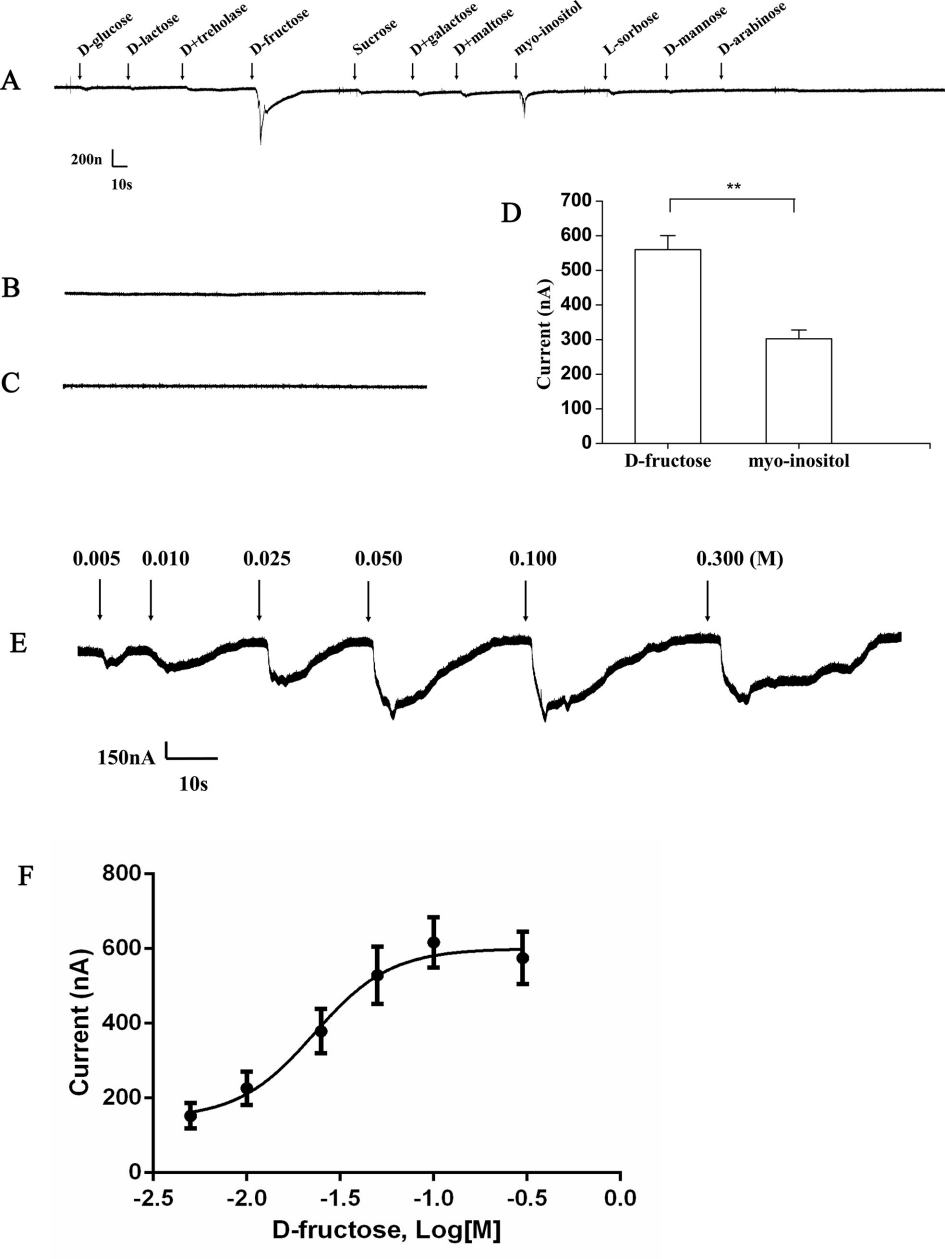
Two-electrode voltage-clamp recordings of *Xenopus* oocytes expressing *TchiGR43a* isolated in the present study. (A) Inward current responses of the oocytes expressing *TchiGR43a* in response to 0.100 M solution of the 11 sugars. (B) *Xenopus* oocytes with no injection. (C) *Xenopus* oocytes injected with ddH_2_O. (D) Inward current responses of the oocytes expressing *TchiGR43a* in response to 0.100 M solution of D-fructose and myo-inositol (mean ± SEM (n = 5)). (E) The oocytes expressing *TchiGR43a* stimulated with a range of D-fructose concentrations. (F) Dose-response curve of the oocytes expressing *TchiGR43a* to D-fructose. EC_50_ = 0.023 M. Bars indicate SEM (n = 6).

We found that the oocytes expressing TchiGR43a showed responses to D-fructose and myo-inositol. There were significant differences between the response to D-fructose and myo-inositol, with a stronger response observed for D-fructose (Fig 5A and 5D). The D-fructose-induced current increased with fructose concentration from 0.005 to 0.300 M (Fig 5E). Based on the dose-response curve, the D-fructose evaluated EC_50_ value was 0.023 M (n = 6) for TchiGR43a (Fig 5F). In the tests, one kind of the control cells were without injected cRNA of *TchiGR43a* and the other kind of the control cells were only injected with ddH_2_O; all of these control cells showed no response to the 11 sugars (Fig 5B and 5C).

## Discussion

Detecting sugars in the living environment is critical for adult Hymenoptera. As a food source, sugars consumed are determined not only at the sensory but also at the physiological level. *T*. *chilonis* showed a behavioural preference response to D-fructose. Our results are consistent with those of other hymenopteran parasitoids such as *Trichogramma japonicum* [41] and *Diadegma semiclausum* [56], indicating that D-fructose could induce feeding behaviour, which might help with the exploitation of fructose as a main food source in nature. As a main sugar in various floral nectars and honeydew, fructose induces a feeding stimulation effect on insects, and this is caused by the palatability and nutritional value of fructose [57].

Feeding with D-fructose significantly prolonged *T*. *chilonis* longevity, as observed in other studies with parasitoids [41, 56, 58]. This finding indicates that D-fructose could be highly attractive to parasitoids and have an important role in their biological activity. Increasing the reproductive potential is crucial for improving the efficiency and ability of parasitoids to control pests. In general, fecundity increase with an increase in life span [59, 42]. In our study, D-fructose increased the longevity of *T*. *chilonis* and also contributed to parasitoid fecundity. In contrast to controls, the fecundity of *T*. *chilonis* feeding on D-fructose almost doubled. The results indicated that diet, to a large extent, affected egg load during the oviposition period, which is consistent with the results from research on the parasitoids *Microplitis mediator* and *Gonatocerus ashmeadi* [42, 60]. The levels of sugars were low in the newly emerged parasitoids, which is supported by previous studies [61, 62]. After emerging, the wasps consumed sugars when D-fructose diets were provided, which revealed that the wasps might effectively utilize D-fructose [43]. Another study also showed that the D-fructose is correlated with reproduction, indicating that an increase in progeny is related with sugar intake in *T*. *chilonis* [41].

Fructose is suitable as a food source that prolongs the longevity of *T*. *chilonis* adults, increases their oviposition and prolongs the oviposition period. This study provided valuable insights for further understanding of *T*. *chilonis* in the field nutrition physiology research, and provided possibilities for the optimal utilization of sugar feedstuffs, for example, planting flowering plants could enhance the activity and efficiency of *Trichogramma* spp. [63–66], and the application of sugar sprays with certain concentration may also increase *Trichogramma* spp. efficiency [67].

The candidate gustatory receptor from *T*. *chilonis*, DmelGR43a, BmorGR9 and the members of a GR43a family from Hymenoptera formed a monophyletic subclade distinct from others (Fig 3). DmelGR43a and BmorGR9 were identified as having the function of sensing fructose, from the phylogenetic tree analysis we speculated that TchiGR43a may also have the same function. These members of the GR43a family from Hymenopteran species, including TchiGR43a, might be evolutionarily homologous with similar mechanisms or modalities for sensing sugars.

Gustatory receptors are in the membrane of gustatory neurons, which are housed in sensilla [4–7]. In this study, the results showed that *TchiGR43a* expressed from the larval stage to the pupal stage, indicating that the gustatory neurons related to *TchiGR43a* might exist from larval to pupal stages; thus, we suggest that some gustatory neurons are persistent larval neurons that form a new system in the pupa and adult [68]. When in the prepupal stage, the expression levels of *TchiGR43a* gene slightly declined, which might be due to the decrease in taste sensilla. Some larval sensilla are lost during metamorphosis and are replaced by new sensilla that originate from imaginal discs [69–71], indicating that gustatory neurons are reorganized in metamorphosis [72]. However, in the early phase of the pupal stage, the expression levels of *TchiGR43a* gene increased, which might be the result of an increase in gustatory neurons. For example, in *Phormia regina*, several hours after pupation some groups of gustatory neurons largely developed, and then, many new gustatory neurons appeared in all tarsal segments and the tibia [73]. Thus, we speculated that during metamorphosis, the increase in gustatory neurons and the production of new gustatory neurons might contribute to the high expression of the gustatory receptor gene—*TchiGR43a*. However, in the later period of the pupal stage, the expression levels of *TchiGR43a* declined, which could be caused by a decrease in gustatory neurons. In previous studies, the apoptosis of sensory neurons is observed at a late pupal stage [68, 72, 73].

In our study, the expression levels of *TchiGR43a* were similar between male and female adults, and we speculated that TchiGR43a not only acted as a sensor of fructose in chemosensory sensilla but also detected internal nutrients in other organs, which is supported by recent reports. In *Drosophila* adults, GR43a is also found in the brain and gut and is sufficient to evaluate nutritious carbohydrates and regulate feeding behaviour [24, 74, 75]. In the gut of *Helicoverpa armigera*, HaGR9 acts as a nutrient sensor to guide digestive processes and to protect from harmful substances [76]. The expression levels of *TchiGR43a* among different developmental stages and different genders suggested the existence of stage-specific and sex-specific gustatory tasks.

In the *Xenopus*-based functional studies, TchiGR43a cells showed response to D-fructose and myo-inositol and no response to the other nine sweet tastants. The response to D-fructose was significantly greater than that to myo-inositol. The results indicated that TchiGR43a was the receptor protein for D-fructose, which is also demonstrated in other *in vitro* studies. For example, DmGr43a of *D*. *melanogaster* and BmGr9 of *B*. *mori* shows a response only to D-fructose but not to other sugar tastants [3]. Similar results were also obtained from *Helicovepa armigera*, in which HarmGR4 were tuned to D-fructose [36]. In the present study, *TchiGR43a*, the orthologous gene of *DmGR43a* and *BmGR9*, showed responses to D-fructose and myo-inositol. Three possibilities might explain these differences. First, because TchiGR43a showed 27% and 28% identity to DmGR43a and BmGR9, respectively, at the amino acid level, the ligand-binding ability might differ. Second, *in vivo*, all the tastants must pass through the pores on the sensillum and diffuse into the lymph, with lymph then conveying the diffused tastants to a dendrite at which the final tastants and concentration may be different compared with the experimental cells that are bathed in tastant solution [8]. Third, the density of receptor proteins may be different in *in vivo* than in a heterologous expression system. However, in previous studies, based on experimental gene knockout and transgene rescue, DmGR43a responded to fructose and sucrose [24]. In *H*. *armigera*, HaGR9 responds to fructose, galactose and maltose [76]. Those studies could support our findings, thus, we speculate that D-fructose receptors share certain similarities and differences among insects, which may be due to differences among species or different ecological conditions and natural habitats. The EC_50_ values of fructose are 0.055 mM for BmGr9 in *B*. *mori* [3], 0.045 M for HarmGr4 in *H*. *armigera* [36] and 0.069 M for AmGr3 in *Apis mellifera* [77], whereas in *T*. *chilonis*, the EC_50_ of fructose was 0.023 M, indicating that the receptor of *T*. *chilonis* are more sensitive than those of lepidopterans.

## Conclusions

We identified, for the first time, the D-fructose receptor in *T*. *chilonis* and verified its function. Behavioural and electrophysiological evidence was provided that *T*. *chilonis* responded to and had a preference for D-fructose. Moreover, the expression of *TchiGR43a* in different developmental stages and genders might also indicate involvement in stage-specific and sex-specific gustatory tasks. Studies on the relationship among D-fructose, D-fructose receptor and physiological behaviours may not only help us understand the underlying molecular mechanism of insect feeding behaviour but also shed light on developing a new strategy in massive production of *T*. *chilonis* for use in biocontrol.

## Supporting information

S1 Table Primers used for 5’ and 3’ RACE, for RT-PCR, and construction of recombinant pCS2+ vectors. S: sense primer; AS: antisense primer. The underlined indicate restriction recognition sites, the italic indicate bases flanking the recognition sequences, and the bold indicate Kozak sequence.
(DOCX)

S1 Dataset The data necessary to replicate the results alongside the manuscript. (XLSX)

## References

[1] Scott K. Taste recognition: food for thought. Neuron. 2005; 48: 455–464. 10.1016/j.neuron.2005.10.015 16269362

[2] Lee Y, Poudel S. Taste sensation in *Drosophila melanoganster*. Hanyang Med Rev. 2014; 34: 130–136. 10.7599/hmr.2014.34.3.130

[3] Sato K, Tanaka K, Touhara K. Sugar-regulated cation channel formed by an insect gustatory receptor. Proc Natl Acad Sci USA. 2011; 108: 11680–11685. 10.1073/pnas.1019622108 21709218 PMC3136286

[4] Stocker RF. The organization of the chemosensory system in *Drosophila melanogaster*: A review. Cell Tissue Res. 1994; 275: 3–26. 10.1007/BF00305372 8118845

[5] Scott K, Brady R Jr, Cravchik A, Morozov P, Rzhetsky A, Zuker C, et al A chemosensory gene family encoding candidate gustatory and olfactory receptors in *Drosophila*. Cell. 2001; 104: 661–673. 10.1016/S0092-8674(01)00263-X 11257221

[6] Amrein H, Thorne N. Gustatory perception and behavior in *Drosophila melanogaster*. Curr Biol. 2005; 15: R673–R684. 10.1016/j.cub.2005.08.021 16139201

[7] Montell C. A taste of the *Drosophila* gustatory receptors. Curr Opin Neurobiol. 2009; 19: 345–353. 10.1016/j.conb.2009.07.001 19660932 PMC2747619

[8] Chyb S, Dahanukar A, Wickens A, Carlson JR. *Drosophila* Gr5a encodes a taste receptor tuned to trehalose. Proc Natl Acad Sci USA. 2003; 2(24): 14526–14530. 10.1073/pnas.2135339100 PMC304113 14523229

[9] Meunier N, Marion-Poll F, Rospars JP, Tanimura T. Peripheral coding of bitter taste in *Drosophila*. J Neurobiol. 2003; 56:139–152. 10.1002/neu.10235 12838579

[10] Dahanukar A, Lei YT, Kwon JY, Carlson JR. Two Gr genes underlie sugar reception in *Drosophila*. Neuron. 2007; 56: 503–516. 10.1016/j.neuron.2007.10.024 17988633 PMC2096712

[11] Poudel S, Kim Y, Kwak J, Jeong S, Lee Y. Gustatory receptor 22e is essential for sensing chloroquine and strychnine in *Drosophila melanogaster*. Insect Biochem Mol Biol. 2017; 88: 30–36. 10.1016/j.ibmb.2017.07.007 28751111

[12] Clyne PJ, Warr CG, Carlson JR. Candidate taste receptors in *Drosophila*. Science. 2000; 287: 1830–1834. 10.1126/science.287.5459.1830 10710312

[13] Zhang HJ, Anderson AR, Trowell SC, Luo AR, Xiang ZH, Xia QY. Topological and functional characterization of an insect gustatory receptor. PLoS ONE. 2011; 6: e24111 10.1371/journal.pone.0024111 21912618 PMC3163651

[14] Hill CA, Fox AN, Pitts RJ, Kent LB, Tan PL, Chrystal MA, et al G proten-coupled receptors in *Anopheles gambiae*. Science. 2002; 298 (5591): 176–178. 10.1126/science.1076196 12364795

[15] Robertson HM, Warr CG, Carlson JR. Molecular evolution of the insect chemoreceptor gene superfamily in *Drosophila melanogaster*. Proc Natl Acad Sci USA. 2003; 100(Suppl 2): 14537–14542. 10.1073/pnas.2335847100 14608037 PMC304115

[16] Wanner KW, Robertson HM. The gustatory receptor family in the silkworm moth *Bombyx mori* is characterized by a large expansion of a single lineage of putative bitter receptors. Insect Mol Biol. 2008; 17 (6): 621–629. 10.1111/j.1365-2583.2008.00836.x 19133074

[17] Grosse-wilde E, Kuebler LS, Bucks S, Vogel H, Wicher D, Hansson BS. Antennal transcriptome of *Manduca sexta*. Proc Natl Acad Sci USA. 2011; 108(18): 7449–7454. 10.1073/pnas.1017963108 21498690 PMC3088587

[18] Smith CD, Zimin A, Holt C, Abouheif E, Benton R, Cash E, et al Draft genome of the globally widespread and invasive Argentine ant (*Linepithema humile*). Proc Natl Acad Sci USA. 2011; 108 (14): 5673–5678. 10.1073/pnas.1008617108 21282631 PMC3078359

[19] Dahanukar A, Foster K, van der Goes van Naters WM, Carlson JR. A Gr receptor is required for response to the sugar trehalose in taste neurons of *Drosophila*. Nat Neurosci. 2001; 4: 1182–1186. 10.1038/nn765 11704765

[20] Jiao Y, Moon SJ, Wang X, Ren Q, Montell C. Gr64f is required in combination with other gustatory receptors for sugar detection in *Drosophila*. Curr Biol. 2008; 18: 1797–1801. 10.1016/j.cub.2008.10.009 19026541 PMC2676565

[21] Slone J, Daniels J, Amrein H. Sugar receptors in *Drosophila*. Curr Biol. 2007; 17(20): 1809–1816. 10.1016/j.cub.2007.09.027 17919910 PMC2078200

[22] Jiao Y, Moon SJ, Montell C. A *Drosophila* gustatory receptor required for the responses to sucrose, glucose, and maltose identified by mRNA tagging. Proc Natl Acad Sci USA. 2007; 104(35): 14110–14115. 10.1073/pnas.0702421104 17715294 PMC1955822

[23] Miyamoto T, Chen Y, Slone J, Amrein H. Identification of a *Drosophila* glucose receptor using Ca^2+^ imaging of single chemosensory neurons. PloS ONE. 2013; 8(2): e56304–25. 10.1371/journal.pone.0056304 23418550 PMC3571953

[24] Miyamoto T, Slone J, Song X, Amrein H. A fructose receptor functions as a nutrient sensor in the *Drosophila* brain. Cell. 2012; 151: 1113–1125. 10.1016/j.cell.2012.10.024 23178127 PMC3509419

[25] Sung HY, Jeong YT, Lim JY, Kim H, Oh SM, Hwang SW, et al Heterogeneity in the *Drosophila* gustatory receptor complexes that detect aversive compounds. Nat Commun. 2017; 8(1). 10.1038/s41467-017-01639-5 PMC5684318 29133786

[26] Lee Y, Moon SJ, Montell C. Multiple gustatory receptors required for the caffeine response in *Drosophila*. Proc Natl Acad Sci. 2009; 106: 4495–4500. 10.1073/pnas.0811744106 19246397 PMC2657413

[27] Poudel S, Kim Y, Kim YT, Lee Y. Gustatory receptors required for sensing umbelliferone in *Drosophila melanogaster*. Insect Biochem Mol Biol. 2015; 66: 110–118. 10.1016/j.ibmb.2015.10.010 26524963

[28] Poudel S, Lee Y. Gustatory receptors required for avoiding the toxic compound coumarin in *Drosophila melanogaster*. Mol cells. 2016; 39: 310 10.14348/molcells.2016.2250 26912085 PMC4844937

[29] Thorne N, Chromey C, Bray S, Amrein H. Taste perception and coding in *Drosophila*. Curr Biol. 2004; 14: 1065–1079. 10.1016/j.cub.2004.05.019 15202999

[30] Wang Z, Singhvi A, Kong P, Scott K. Taste representations in the *Drosophila* brain. Cell. 2004; 117: 981–991. 10.1016/j.cell.2004.06.011 15210117

[31] Lee Y, Kang MJ, Shim J, Cheong CU, Moon SJ, Montell C. Gustatory receptors required for avoiding the insecticide L-canavanine. J Neurosci. 2012; 32: 1429–1435. 10.1523/JNEUROSCI.4630-11.2012 22279227 PMC3356580

[32] Lee Y, Moon SJ, Wang Y, Montell C. A *Drosophila* gustatory receptor required for strychnine sensation. Chem Senses. 2015; 40: 525–533. 10.1093/chemse/bjv038 26187906 PMC4580539

[33] Shim J, Lee Y, Jeong YT, Kim Y, Lee MG, Montell C, Moon SJ. The full repertoire of *Drosophila* gustatory receptors for detecting an aversive compound. Nat Commun. 2015; 6: 8867 10.1038/ncomms9867 26568264 PMC4660205

[34] Zhang YF, Huang LQ, Ge F, Wang CZ. Tarsal taste neurons of *Helicoverpa assulta* (Guenee) respond to sugars and amino acids, suggesting a role in feeding and oviposition. J Insect Physiol. 2011; 57: 1332–1340. 10.1016/j.jinsphys.2011.06.009 21771596

[35] Robertson HM, Wanner KW. The chemoreceptor superfamily in the honey bee, *Apis mellifera*: Expansion of the odorant, but not gustatory, receptor family. Genome Res. 2006; 16: 1395–1403. 10.1101/gr.5057506 17065611 PMC1626641

[36] Jiang XJ, Ning C, Guo H, Jia YY, Huang LQ, Qu MJ, et al A Gustatory Receptor Tuned to D-fructose in Antennal Sensilla chaetica of *Helicoverpa armigera*. Insect Biochem Mol Biol. 2015; 60: 39–46. 10.1016/j.ibmb.2015.03.002 25784630

[37] Jung JW, Park KW, Ahn YJ, Kwon HW. Functional characterization of sugar receptors in the western honeybee, *Apis mellifera*. J Asia-Pac Entomol. 2015; 18(1): 19–26. 10.1016/j.aspen.2014.10.011

[38] Baker HG. Sugar concentrations in nectars from hummingbird flowers. Biotropica. 1975; 7: 37–41. 10.2307/2989798

[39] Leatemia JA, Laing JE, Corrigan JE. Production of exclusively male progeny by mated, honey-fed *Trichogramma minuturn* Riley (Hym., Trichogrammatidae). J Appl Entomol. 1995; 119(1–5): 561–566. 10.1111/j.1439-0418.1995.tb01336.x

[40] Wäckers FL. Gustatory Response by the Hymenopteran Parasitoid *Cotesia glomeratato* a Range of Nectar and Honeydew Sugars. J. Chem. Ecol. 1999; 25(12):2863–2877. 10.1023/a:1020868027970

[41] Tian JC, Wang GW, Romeis J, Zheng XS, Xu HX, Zang LS, et al Different Performance of Two *Trichogramma* (Hymenoptera: Trichogrammatidae) Species Feeding on Sugars. Environ. Entomol. 2016; 45(5): 1316–1321. 10.1093/ee/nvw106 27542400

[42] Luo SP, Li JC, Liu XX, Lu ZY, Pan WL, Zhang QW, et al Effects of six sugars on the longevity, fecundity and nutrient reserves of *Microplitis mediator*. Biol. Control. 2010; 52(1):51–57. 10.1016/j.biocontrol.2009.09.002

[43] Wäckers FL. A comparison of nectar and honeydew sugars with respect to their utilization by the Hymenopteran parasitoid *Cotesia glomerata*. J Insect Physiol. 2001; 47, 1077–1084. 10.1016/S0022-1910(01)00088-9 11472770

[44] Wolcott GN. The requirements of parasites for more than hosts. Science. 1942; 96 (2492): 317–8. 10.1126/science.96.2492.317 17751367

[45] Sahragard A, Jervis MA, Kidd NAC. Influence of host availability on rates of oviposition and host-feeding, and on longevity in *Dicondylus indianus Olmi* (Hymenoptera., Dryinidae), a parasitoid of the rice brown planthopper, *Nilaparata lugens* Stal (Hemiptera., Del-phacidae). J Appl Entomol. 1991; 112: 153–162. 10.1111/j.1439-0418.1991.tb01041.x

[46] Zhan GX, Liang GW. Research and application of *Trichogramma* in China. Acta Agric Jiangxi. 1999; 11: 39–46. 10.19386/j.cnki.jxnyxb.1999.02.009

[47] Shi ZS, Chen HS, Qin ZQ, Guo Q, Bi DJ, Jiang QM, et al Population Dynamics of Borers and Its Control Effect Evaluation by Using *Trichogramma chilonis* Ishii in Chongzuo Cane Area. Chinese Journal of Biological Control. 2018; 34(5)656–662. 10.16409/j.cnki.2095-039x.2018.05.002

[48] Chen LL, Lin CM, Xie YL, Qin BR, Wang HS. Experiment on rice leaf folder control by releasing *Tirchogramma Chilonis*. Journal of Guangxi Agriculture, 2016.2016; 31(4). 10.3969/j.issn.1003-4374.2016.04.005

[49] Fatima B, Ashraf M, Ahmad N, Suleman N. Mass production of Trichogramma chilonis: an economical and advanced technique. The BCPC Conference: Pests and diseases, Volumes 1 and 2. Proceedings of an international conference held at the Brighton Hilton Metropole Hotel, Brighton, UK, 18–21 November 2002.

[50] Kazuki M, Masahiro K. Effects of host-egg age on the parasitism by *Trichogramma chilonis* Ishii (Hymenoptera: Trichogrammatidae), an egg parasitoid of the diamondback moth. Appl Entomol Zool. 1998; 33(2): 219–222. 10.1303/aez.33.219

[51] Dadmal SM, Pujari AJ, Satpute NS. Influence of short term exposure to different temperatures on key biological parameters of *Trichogramma chilonis* Ishii under laboratory conditions. J Biol Control. 2010; 24 (1): 8–12. 10.18311/jbc/2010/3558

[52] Liu JB, Wu H, Yi JQ, Song ZW, Li DS, Zhang GR. Transcriptome characterization and gene expression analysis related to chemoreception in *Trichogramma chilonis*, an egg parasitoid. Gene.2018; 678: 288–301. 10.1016/j.gene.2018.07.065 30107229

[53] Weiss LA, Dahanukar A, Kwon JY, Banerjee D, Carlson JR. The molecular and cellular basis of bitter taste in *Drosophila*. Neuron. 2011; 69(2):258–272. 10.1016/j.neuron.2011.01.001 21262465 PMC3033050

[54] Livak KJ, Schmittgen TD. Analysis of relative gene expression data using real-time quantitative PCR and the 2^−ΔΔct^, method. Methods. 2001; 25 (4): 402–408. 10.1006/meth.2001.1262 11846609

[55] Huang YC, Yi DW, Song ZW, Li DS, Zhang GR. The individual development of *Trichogramma chilonis* on *Corcyra cephalonica* eggs. J Env Ent. 2016; 38 (3): 457–462. 10.3969/j.issn.1674-0858.2016.03.1

[56] Winkler K, Wäckers FL, Stingli A, Jcvan L. *Plutella xylostella* (diamondback moth) and its parasitoid *Diadegma semiclausum* show different gustatory and longevity responses to a range of nectar and honeydew sugars. Entomol Exp Appl. 2010; 115: 187–192. 10.1111/j.1570-7458.2005.00254.x

[57] Romeis J, Wäckers FL. Feeding responses by female *Pieris brassicae* butterflies to carbohydrates and amino acids. Physiol Entomol. 2000; 25(3): 247–253. 10.1046/j.1365-3032.2000.00188.x

[58] Wäckers FL. Gustatory response by the hymenopteran parasitoid *Cotesia glomerata* to a range of nectar and honeydew sugars. J Chem Ecol. 1999; 25(12): 2863–2877. 10.1023/A:1020868027970

[59] Stapel JO, Cortesero AM, Moraes CMD, Tumlinson JH, Lewis WJ. Extrafloral nectar, honeydew, and sucrose effects on searching behavior and efficiency of *Microplitis croceipes* (Hymenoptera: Braconidae) in cotton. Environ Entomol. 1997; 26(3): 617–623. 10.1093/ee/26.3.617

[60] Irvin NA, Hoddle MS, Castle SJ. The effect of resource provisioning and sugar composition of foods on longevity of three *Gonatocerus* spp. egg parasitoids of *Homalodisca vitripennis*. Biol Control. 2007; 40(1): 69–79. 10.1016/j.biocontrol.2006.09.005

[61] Olson DM, Fadamiro H, Lundgren JG, Heimpel GE. Effects of sugar-feeding on carbohydrate and lipid metabolism in a parasitoid wasp. Physiol Entomol. 2000; 25: 17–26. 10.1046/j.1365-3032.2000.00155.x

[62] Chen L, Fadamiro HY. Comparing the effects of five naturally occurring monosaccharide and oligosaccharide sugars on longevity and carbohydrate nutrient levels of a parasitic phorid fly, *Pseudacteon tricuspis*. Physiol Entomol. 2006; 31: 46–56. 10.1111/j.1365-3032.2005.00484.x

[63] Landis DA, Wratten SD, Gurr GM. Habitat management to conserve natural enemies of arthropod pests in agriculture. 2000; Annu. Rev. Entomol. 45: 175–201. 10.1146/annurev.ento.45.1.175 10761575

[64] Romeis J, Babendreier D, Wäckers FL, Shanower TG. Habitat and plant specificity of *Trichogramma* egg parasitoids—underlying mechanisms and implications. Basic Appl. Ecol. 2005; 6: 215–236. 10.1016/j.baae.2004.10.004

[65] Gurr GM, Liu J, Read DM, Catindig JLA, Cheng JA, Lan L, et al Parasitoids of Asian rice planthopper (Hemiptera: Delphacidae) pests and prospects for enhancing biological control by ecological engineering. Ann. Appl. Biol. 2011; 158: 149–176. 10.1111/j.1744-7348.2010.00455.x

[66] Zhu P, Wang G, Zheng X, Tian J, Lu Z, Heong KL, et al Selective enhancement of parasitoids of rice Lepidoptera pests by sesame (*Sesamum indicum*) flowers. BioControl. 2015; 60: 157–167. 10.1007/s10526-014-9628-1

[67] Tena A, Pekas A, Cano D, Wäckers FL, Urbaneja A. Sugar provisioning maximizes the biocontrol service of parasitoids. J. Appl. Ecol. 2015; 52: 795–804. 10.1111/1365-2664.12426

[68] Gendre N, Lüer K, Friche S, Grillenzoni N, Ramaekers A. Technau GM, Stocker RF. Integration of complex larval chemosensory organs into the adult nervous system of *Drosophila*. Development. 2004; 131(1): 83–92. 10.1242/dev.00879 14645122

[69] Levine RB, Morton DB, Restifo LL. Remodeling of the insect nervous system. Curr Opin Neurobiol. 1995; 5: 28–35. 10.1016/0959-4388(95)80083-2 7773002

[70] Truman JW. Metamorphosis of the insect nervous system In Metamorphosis: Postembryonic Reprogramming of Gene Expression in Amphibian and Insect Cells (ed. Gilbert L. I., Tata J. R. and Atkinson B. G.), San Diego: Academic Press; 1996 pp. 283–320. 10.1016/B978-012283245-1/50010-5

[71] Tissot M, Stocker RF. Metamorphosis in *Drosophila* and other insects: the fate of neurons throughout the stages. Prog Neurobiol. 2000; 62: 89–111. 10.1016/S0301-0082(99)00069-6 10821983

[72] Eichmüller S, Schäfer S. Sensory neuron development revealed by taurine immunocytochemistry in the honeybee. J Comp Neurol. 2010; 352(2): 297–307. 10.1002/cne.903520211 7721996

[73] Lakes R, Pollack GS. The development of the sensory organs of the legs in the blowfly, *Phormia regina*. Cell Tissue Res. 1990; 259(1): 93 10.1007/BF00571434 2297787

[74] Park JH, Kwon JY. Heterogeneous expression of *Drosophila* gustatory receptors in enteroendocrine cells. PloS ONE. 2011; 6(12): e29022 10.1371/journal.pone.0029022 22194978 PMC3237578

[75] Miyamoto T, Amrein H. Diverse roles for the *Drosophila* fructose sensor Gr43a. Fly. 2014; 8 (1): 19–25. 10.4161/fly.27241 24406333 PMC3974889

[76] Xu W, Zhang HJ, Alisha AA. Sugar gustatory receptor identified from the foregut of cotton bollworm *Helicoverpa armigera*. J Chem Ecol. 2012; 38(12): 1513–1520. 10.1007/s10886-012-0221-8 23224441 PMC3532720

[77] Takada T, Sasaki T, Sato R, Kikuta S, Inoue MN. Differential expression of a fructose receptor gene in honey bee workers according to age and behavioral role. Arch Insect Biochem Physiol. 2018; 97(2): e21477 10.1002/arch.21437 29194737

