# Supplementary material for: Identification and functional characterization of D-fructose receptor in an egg parasitoid, Trichogramma chilonis
Source: PLoS One. 2019 Jun 19;14(6):e0217493. doi: 10.1371/journal.pone.0217493 (PMC6583964; doi:10.1371/journal.pone.0217493)
Supplement: S1 Table — S: sense primer; AS: antisense primer. The underlined indicate restriction recognition sites, the italic indicate bases flanking the recognition sequences, and the bold indicate Kozak sequence. (DOCX) [file pone.0217493.s001.docx]

Supplementary Table

Primers used for 5’ and 3’ RACE, for RT-PCR, and construction of recombinant pCS2+ vectors

| Gene  (Accession number) | Primer sequences (5’-3’) | S/AS |
| --- | --- | --- |
| For 5’ and 3’ RACE | | |
| TchiGR43a (MH816967) | TCGGTCTCCACGGCATCCT | S |
|  | TCATCACGCCCTACTTCCTCTACAA | S |
|  | CGCAGACGCTCGGATGT | AS |
|  | TGTTCTCCCAGCCGTTGC | AS |
| UPM | CTAATACGACTCACTATAGGGCAAGCAGTGGTATCAACGCAGAGT |  |
|  | CTAATACGACTCACTATAGGGC |  |
| NUP | AAGCAGTGGTATCAACGCAGAGT |  |
| For construction of recombinant pCS2+ vectors | | |
| TchiGR43a | CGGAATTC**GCCACC**ATGGGGATTCGTCGGCACGCGAT | S |
|  | GCTCTAGATCATCTGACTTTGAAGGTAATATTT | AS |
| For qRT-PCR | | |
| TchiGR43a | GCAACGGCTGGGAGAAC | S |
|  | CGCAGACGCTCGGATGT | AS |

S: sense primer; AS: antisense primer. The underlined indicate restriction recognition sites, the italic indicate bases flanking the recognition sequences, and the bold indicate Kozak sequence.
